# Potential therapeutic role of Tridham in human hepatocellular carcinoma cell line through induction of p53 independent apoptosis

**DOI:** 10.1186/1472-6882-13-323

**Published:** 2013-11-21

**Authors:** Ravindran Jaganathan, Vijaya Ravinayagam, Sachdanandam Panchanadham, Shanthi Palanivelu

**Affiliations:** 1 Department of Pathology, Dr. ALM Post Graduate Institute of Basic Medical Sciences, University of Madras, Taramani Campus, Chennai, Tamilnadu 600113, India; 2 Department of Medical Biochemistry, Dr. ALM Post Graduate Institute of Basic Medical Sciences, University of Madras, Taramani Campus, Chennai, Tamilnadu 600113, India

**Keywords:** Hepatocellular carcinoma (HCC), Tridham, p53, Apoptosis, Mitochondrial membrane potential (MMP)

## Abstract

**Background:**

Hepatocellular carcinoma (HCC) is the third leading cause of cancer deaths reported worldwide. The incidence is higher in Asia and Africa, where there is greater endemic prevalence of hepatitis B and C. The devastating outcome of cancer can be minimized only by the use of potent therapeutic agents. Tridham (TD) has been acknowledged since olden days for its wide spectrum of biological properties and was used by traditional practitioners of Siddha and other indigenous systems of medicine. The present study aims at investigating the mechanistic action of TD by assessing the antiproliferative and pro-apoptotic effects on human hepatocellular carcinoma cell line (Huh7).

**Methods:**

Cell viability and apoptosis assay using MTT analysis and trypan blue staining, DAPI staining, DNA fragmentation, cell cycle analysis, mitochondrial membrane potential, real-time reverse transcription-polymerase chain reaction, western blotting and immunofluorescence staining were determined in Huh7 cells.

**Results:**

Viability studies of TD treated Huh7 cells showed an inhibition in cell growth in time and dose dependent manner. Chromatin condensation, DNA fragmentation and apoptotic bodies, which are structural changes characteristic of apoptosis, were found following TD treatment of Huh7 cells. DAPI staining and agarose gel electrophoresis confirmed the induction of apoptosis by TD. Cell cycle analysis of Huh7 cells treated with TD exhibited a marked accumulation of cells in the sub-G1 phase of the cell cycle in a dose dependent manner. Immunofluorescent staining for Ki-67 showed a higher level of expression in untreated cells as compared to TD treated cells. We observed a significant loss in the mitochondrial membrane potential and the release of cytochrome c into the cytosol in TD treated cells. Down regulation of Bcl-2, up regulation of Bax and Bad as well as activation of caspases-3 and 9 were also observed. The p53 gene expression was found to be unaltered in TD treated cells.

**Conclusion:**

These results suggest that TD induces apoptosis of Huh7 cells through activation of Bax and triggered caspase cascade, independent of p53 function. This study throws light on the mechanistic action of TD in triggering apoptosis in Huh 7 cells.

## Background

Cancer, a life-threatening health problem, is one of the leading causes of death world-wide [1]. Cancer is a hyper proliferative disease that can be classified into three distinct phases: initiation, promotion and progression. Hepatocellular Carcinoma (HCC) is the third most common cause of cancer-related deaths reported world-wide. The incidence of HCC ranges from 10 cases per 100 000 population in North America and Western Europe to 50–150 cases per 100 000 population in parts of Africa and Asia [2]. HCC is more common among the men of Asian region with a lower prevalence in women. The key factors related to the progression of HCC are hepatitis, especially hepatitis B & C, aflatoxin exposure, alcohol consumption and iron overload [3]. As malignant cells defy apoptotic stimuli, a competent way of designing anti-cancer agents is to endow them with the ability to combat tumor cells through modulation of apoptosis [4,5].

Apoptosis or programmed cell death occurs normally during development and aging. Apoptosis also acts as a homeostatic mechanism which maintains cell populations in tissues [6,7]. Caspase-9 is the primary caspase which plays a central role in the mitochondrial apoptotic pathway. Activation of caspase 9 is associated with changes in the permeability of the outer mitochondrial membrane and the collapse of membrane potential. The latter results in release of cytochrome c and activation of downstream effector caspases such as caspase-3 [8,9]. As a key tumor suppressor, p53 plays a critical role in tumor prevention. p53, a checkpoint control protein, maintains genomic integrity and is involved in cell-cycle control. The p53 gene has been found to be over expressed in many types of human malignancies including HCC [10,11]. In the intrinsic pathway of apoptosis, Bad, a pro-apoptotic protein facilitates the induction of apoptosis by two other pro-apoptotic proteins in the Bcl-2 family, Bax and Bak. The latter primarily cause mitochondrial outer membrane permeabilization (MOMP) [12], resulting in the diffusion of proteins of the intermembrane space into the cytosol. Cytochrome c is thereby released into the cytoplasm. This event triggers a biochemical cascade resulting in the activation of caspase proteases which leads to apoptotic cell death.

Current modalities of cancer therapy such as radiation, chemotherapy, immunosuppression and surgery are marked by high morbidity and mortality rate. The results of currently available modalities of treatment for HCC are dismal, especially when the cancer has spread beyond the liver. This has created an imperative need for the development of novel treatment strategies. The most effective and safest stratagem is to prevent, arrest or reverse the cellular and molecular processes of carcinogenesis. This can be achieved by the use of dietary bio factors, phytochemicals and even whole plant extracts [13]. Herbal medicines are now considered as a part of Complementary and Alternative medicine (CAM) which is defined as a group of diverse medical and health care systems, practices and products that are not presently considered to be part of conventional Western medicine. The therapeutic activities of plants have been attributed to the presence of secondary metabolites [14]. Herbal medicines are gaining popularity due to their potent antioxidant activity, minimal side effects and economic viability [15]. Among the different types of herbal medicine, Siddha medicine is a form of traditional medicine, used for nearly 10,000 years, by the people of Southern India. This ancient Siddha System of Medicine possesses distinctive features with respect to its physiology, pathology, pharmacology and therapeutics [16].

Tridham (TD) is a combination of three plant ingredients including seed coats of *Terminalia chebula* (*T. chebula*), fruits of *Elaeocarpus ganitrus* (*E. ganitrus*) and leaves of *Prosopis cineraria* (*P. cineraria*) in equal proportions. TD is used by traditional practitioners of Siddha medicine and has been scientifically proven to have potent anticancer and antioxidant properties against Aflatoxin B1 induced rat hepatocellular carcinoma [17]. *T. chebula* belongs to the family *Combretaceae* and is commonly found in the deciduous forests, teak forests and on the dry slopes of the Indian subcontinent. Extensive studies are available on *T. chebula* for its wide spectrum of biological properties such as anti-bacterial [18], anti-fungal [19], anti - diabetic [20], antioxidant [21,22], anti-cancer [23], hepatoprotective [24] and anti-mutagenic [25]. *T. chebula* extract has growth inhibitory and cytotoxic effects on several human cancer cell lines [22]. *T. chebula* has been reported to inhibit growth of HCT-15 and HepG2 cells [26]. Presence of several phytocomponents namely, gallic acid, ellagic acid; tannic acid; β-sitosterol; ethylgallate; chebulic acid and mannitol were observed in phytochemical analysis of *T. chebula. T. chebula* has been found to be one of the richest sources of ascorbic acid [25,27]. Quercetin, found in *T. chebula* (a component of TD), inhibits cell invasion and induces apoptosis in the HepG2 cell line [28]. *E. ganitrus*, which belongs to the *Elaeocarpaceae* family, is used in Ayurveda for treating various diseases such as mental illness, epilepsy, asthma, hypertension, anti-aging, arthritis, hysteria, cough and hepatic diseases [29]. Potentially rich anti-cancer agents such as gallic acid and ellagic acid have been reported to be present in *E. ganitrus*[30,31]. Palmitic acid, reported to be present in *E. ganitrus* (another component of TD), induces apoptosis through mitochondria-mediated pathway by regulating Bcl-2/Bax ratio [32,33]. *P. cineraria* is considered to have a number of medicinal effects such as anti-helminthic, anti-cancer, anti-bacterial, anti-fungal, anti-viral, anti-diabetic and many other pharmacological properties [34]. The smoke of the leaves is considered good for eye ailments. Leaf paste of *P. cineraria* is applied on boils, blisters and mouth ulcers in livestock Leaf infusion is used to treat open sores on the skin [35]. Several bioactive compounds such as flavonoids, alkaloids, diketones, phenolic contents, free amino acids, patulitrin, spicigerin, prosogerin A, B, C, D, lipids, β-sitosterol, sugars and vitamins have been isolated from *P. cineraria*[34,36].

Preceding studies exemplify that each of the three components of TD has their proven medicinal value individually and the amalgamation of these is likely to augment their antioxidant activity. Earlier studies in our laboratory revealed that the aqueous extract of TD had anti-oxidant and free radical scavenging effects. TD was also found to inhibit cell growth and induce apoptosis in the human hepatocellular carcinoma cell line, HepG2 [37]. However, the underlying mechanism of anticancer action of TD is unclear. In addition to the presence of various constituents in TD, gallic acid was isolated with high purity from TD [37]. Gallic acid has been reported to exhibit cytotoxicity in various cancer cell lines including HepJ5, a liver cancer cell line [38].

The present study aims at determining the mechanism of cell death elicited by TD. We have used morphological and molecular investigations with respect to mitochondrial mediated apoptosis in an attempt to explore the mechanistic actions of TD on human hepatocellular carcinoma cell line (Huh7).

## Methods

### Chemicals and reagents

Ethidium bromide, propidium iodide (PI), dimethyl sulfoxide (DMSO), 3-(4,5-dimethylthiazol-2-yl)-2,5-diphenyltetrazolium bromide (MTT), trypan blue, 4,6-diamidino-2-phenylindole dihydrochloride (DAPI) and nuclear staining with Hoechst 33258 were purchased from Sigma Chemical Co., USA. Trypsin–EDTA, fetal bovine serum (FBS), antibiotics–antimycotics, Dulbecco’s modified Eagle’s medium (DMEM) and phosphate buffered saline (PBS) were purchased from Gibco, Canada. Polyvinylidine difluoride (PVDF) membrane was purchased from Millipore, USA. Primary antibodies against Bax, Bcl-2, p53, cytochrome c, caspase-9, and caspase-3 were purchased from Santa Cruz Biotechnology (Santa Cruz, CA, USA). Actin antibody was purchased from Sigma–Aldrich Chemicals Pvt. Ltd (USA). The secondary antibodies, horseradish peroxidase (HRP) conjugated goat anti-mouse IgG and goat anti-rabbit IgG were obtained from Bangalore Genei, Bangalore, India. All other chemicals used were of extra pure analytical grade.

### Cell culture

Human hepatocellular carcinoma cell line (Huh-7) and Chang liver cell line were obtained from Cell Repository at National Centre for Cell Sciences, Pune, India. Human HCC cell line (Huh-7) was grown in T-25 culture flask containing 1:1 mixture of Dulbecco’s Modified Eagle Medium (DMEM) and Ham’s F12 media supplemented with 10% (v/v) FBS, 1% antibiotics (100 units/ml penicillin, 100 μg/ml streptomycin) and 1 mM pyruvate at 37°C in a humidified atmosphere containing 5% CO_2_.

### Drug preparation

The three ingredients of TD were collected and authenticated botanically and deposited in the herbarium of the Centre for Advanced Studies (CAS) in Botany, University of Madras, Guindy Campus, Chennai, India. Herbarium numbers of ingredients are CASBH-16 (*T. chebula*), CASBH-17 (*E. ganitrus*), and CASBH-18 (*P. cineraria*). The ingredients were washed, air dried in shade and then finely ground. The aqueous extract of TD was prepared in 3:1(v/w) ratio, mixed by using a shaker for 12 h followed by subsequent filtration and the clear filtrate (aqueous extract) was collected in a beaker. The filtrate was then lyophilized under vacuum pressure to yield a powder. The lyophilized extract was stored in airtight containers in a dry dark place [17,37]. TD was dissolved in 1% DMSO [final concentration of the DMSO did not exceed 1% (v/v) and did not affect the cell proliferation] prepared in serum free DMEM medium and filtered by 0.3 mm syringe filter and stored.

### Cell viability assay

To determine the effective dose and time of TD on Huh 7 cell line, the MTT (3-(4, 5-dimethylthiazol-2yl-)-2, 5-diphenyl tetrazolium bromide) assay and Trypan blue staining were employed.

### MTT assay

Approximately, 5 × 10^3^ cells/well were seeded into 96-well tissue culture plates and 24 h after seeding, the medium was changed and cells were treated with various concentrations of TD, ranging from 20 to 200 μg/ml and incubated for 24 and 48 hrs. Ten μl of MTT solution (5 mg/ml in PBS) was added to each well. The plates were wrapped with aluminum foil and incubated for 4 h at 37°C. The purple formazan crystal formed at the bottom of the wells was dissolved with 180 μl DMSO for 20 min. and the absorbance at 570 nm was read on a microplate reader (Sirios, Seac Radim Group, Italy). Cell viability was expressed as the optical density ratio of the treated cells to the control (% control). Percentage of growth inhibition = A_570_ nm of treated cells **/** A_570_ nm of control cells X 100.

### Trypan blue staining

Trypan blue (0.4%) dissolved in PBS was used for cell counting by the method of Kugawa *et al*., [39]. For the determination of cell viability, cells were plated at the density of 5 × 10^4^ cells/well and cultured for 48 h. The medium was replaced with serum-free medium [DMEM medium, supplemented with antibiotics (penicillin100 U/mL, streptomycin10 μg/mL, 1 mmol/L sodium pyruvate)] and the cells were treated with various concentrations of TD (20–200 μg/ml) for 24, 48 and 72 h and incubated with 1% DMSO as solvent control. The percentage of viability can be calculated as: Percentage of viability = (Number of unstained cells **/** Total number of cells) X 100. For assessing the cell morphology, the cells were observed under phase contrast microscope (Nikon Eclipse-80i, Japan) and photographed.

### Determination of cell and nuclear morphology

The cells (200–300 cells/sample) were grown on 12 mm cover slips and exposed to TD in sub confluent stage for 48 h. The monolayer of cells was washed with PBS and fixed with 3% paraformaldehyde for 10 min at room temperature. The fixed cells were permeabilized with 0.2% Triton X-100 in PBS for 10 min at room temperature and incubated with 0.5 μg/ml of DAPI for 5 min. The apoptotic nuclei (intensely stained, fragmented nuclei and condensed chromatin) were viewed under a fluorescent microscope (Nikon Eclipse-80i, Japan) with an excitation at 359 nm and emission at 461 nm wavelengths.

### Measurement of mitochondrial membrane potential

Huh7 cells (100–200 cells/sample) were grown on 12 mm × 12 mm cover slips placed in each well of a six-well plate and exposed to TD (50 and 100 μg/ml) in sub confluent stage for 48 h. After 48 h, the cover slips containing cells were washed with PBS and fixed with methanol for 10 min at room temperature. The fixed cells were permeabilized with 0.2% Triton X-100 in PBS for 10 min at room temperature and incubated with 5 μg/ml of Rhodamine-123 for 30 min at 37°C. This dye is taken up by the mitochondria forming aggregates and exhibits intense red fluorescence. The stained cells were observed under fluorescent microscope (Nikon Eclipse-80i, Japan) with an excitation at 488 nm and emission at 525 nm wavelengths.

### DNA gel electrophoresis

Agarose gel electrophoresis was carried out for the analysis of DNA fragmentation by the method of Yokozawa and Dong, 2001 [40]. The DNA from Huh7 control cells and treated cells were isolated following the manufacturer’s instructions provided by the kit (Bangalore Genei) and dissolved in TE buffer. The DNA samples were electrophoresed on 1.2% Agarose gel using TBE buffer at 40 V for 3 h. Then, the gel was stained with Ethidium bromide (EtBr) and viewed under UV trans illuminator and photographed.

### Western blot analysis

The cells were lysed using RIPA buffer containing 1× protease inhibitor cocktail following which protein concentrations were measured using Lowry’s method [41]. Cell lysates (20–50 μg) were electrophoresed in 12% SDS polyacrylamide gel and then transferred into PVDF membranes. Membranes were blocked with blocking solution (2% BSA in PBS) for 1 h at 37°C followed by incubation with primary antibodies against Bcl-2, Bax, Caspase 9, Caspase 3, cytochrome c and p53 in tris-buffered saline. After being washed, the membranes were incubated with HRP conjugated goat-anti mouse IgG (1:5000) or HRP conjugated goat-anti rabbit IgG (1:5000). Protein bands were detected using chemiluminescence system (ECL Kit) and quantified using Chemi Doc XRS Imaging System, Bio-Rad (USA).

### Determination of caspase-3 activity

Caspase 3 activity was assayed in Huh7 cells by using caspase 3 colorimetric kit (Biovision USA). About 50 μg of protein lysate was taken in an ELISA reader plate, 50 μl of 2X reaction buffer [containing 10 mM dithiothreitol (DTT)] was added to each sample and 5 μl of the 4 mm DEVD (a synthetic tetra peptide, ASP-Glu-Val-Asp) –pNA (p-nitroanilide) substrate was also added and incubated at 37°C for 24 h. The samples were read at 400 nm in an ELISA reader. The absorbance was calculated and expressed in fold change compared to that of control and plotted in a graph.

### Real-time PCR analysis

mRNA expressions of p53, Bcl-2, Bax and Bad were studied by the method of comparative Real time PCR. The total RNA was isolated by using Tri reagent (Invitrogen). About 1 μg of total RNA was reverse transcribed from each of the samples using a commercial iScript cDNA synthesis kit, Bio-Rad, USA. RT-PCR was carried out in MX3000p PCR system (Stratagene, Europe) using Kapa sybr green fast pcr master mix PCR kit. The primers used for p53: forward primer: 5′-GTC TACCTCCCGCCATAA-3′, reverse primer: 5′-CATCTCCCAAACATCCCT-3′(206 bp); Bcl-2 forward primer: 5′-TTGTTCAAACGGGATTCACA-3′, reverse primer: 5′-GAG CAAGTGCAGCCACAATA-3′ (331 bp); Bax forward primer: 5′-GCTGGACATTGGACTTCCTC-3′, reverse primer: 5′-CTCAGC CCATCTTCTTCCAG-3′ (168 bp); Bad forward primer: 5′- CCTCAGGCCTATGCAAAA AG-3′, reverse primer: 5-AAACCCAAAACTTCCGAT GG-3′ (120 bp); GAPDH forward primer: 5′-CGACCACTTTGTCAAGCTCA-3′, reverse primer: 5′-CCCCTCTTCAAGGGGTCTAC-3′ (238 bp). Real time PCR was performed at 95°C for 10 min, followed by 40 cycles (94°C for 30 s; 55°C for 30 s) of denaturation, annealing and extension. All products generated during the PCR amplification reaction were melted at 95°C, then annealed at 55°C and subjected to gradual increases in temperature changes. Fluorescence data were collected until the reaction reached 95°C. The result is a plot of raw fluorescence data units versus-temperature. At every cycle, the fluorescence emitted was calculated and the PCR cycle at which fluorescence measured by the instrument was determined. The threshold cycle (ct) is inversely proportional to the log of the initial copy number.

### Cell cycle analysis

Huh7 cells were grown in T25 flask. On reaching 80%, confluent cells were kept for overnight starvation by adding plain DMEM medium without FBS. On the day of treatment, TD (20–200 μg/ml) was added to the cells, incubated for indicated time periods and then each sample was harvested and fixed in 70% ethanol for 10 h. After fixation, cells were washed with PBS, treated with 3.3 μg/ml of DNase-free RNase (Roche Applied Science, Indianapolis, Indiana) for 20 mins at room temperature and stained with 50 μg/ml of propidium iodide (PI) in 0.1 M sodium citrate buffer (pH 7.4) for 30 mins at 4°C. Stained cells were then analyzed using flow cytometer (FACS Calibur; BD Biosciences, San Jose, California). Data were analyzed using Cell Quest Software (Becton Dickinson, Mountain View, CA, USA).

### Immunofluorescence staining

Immunocytochemical staining was performed as described by the manufacturer’s instructions. Cells were seeded onto glass coverslips in 24 well tissue culture plates and stained with antibody to detect Ki67 expression and the nuclear marker Hoechst 33258 stain and observed under an Advanced Fluorescence Microscope (NIKON 80i, Tokyo, Japan).

### Statistical analysis

The values are expressed as mean ± SD. The results were computed statistically (SPSS software package, version 7.5) using one-way analysis of variance (ANOVA). Post hoc testing was performed for inter-group comparison using Student-Newman-Kuel multiple comparison test. Values of p < 0.05 were considered significant.

## Results

### Cytotoxic effect of TD in Huh7 cells

#### MTT assay

**Figure 1:**
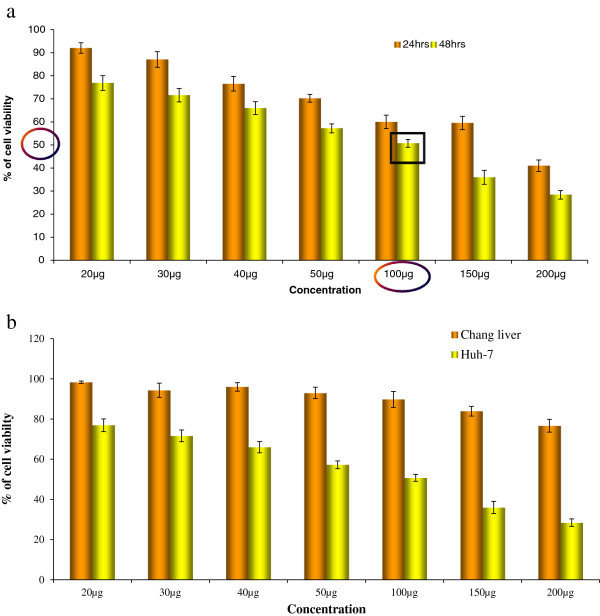
**(a&b): Dose dependent effect of TD on viability of Huh7 and normal liver cells.** Viable cells were determined using the MTT method as described in Materials and methods. The cells were incubated with TD (20 – 200 μg/l) for 24 and 48 h. The results are presented as mean ± SD of data from at least three independent experiments.

The cytotoxic effects of TD in Huh7 cells and Chang liver cells (normal) were measured by using MTT assay. Huh7 cells were incubated in the presence of TD at concentrations of 20 μg/ml, 30 μg/ml, 40 μg/ml, 50 μg/ml, 100 μg/ml, 200 μg/ml for 24 h and 48 h. There was a dose and time dependent decrease in viable cells after 24 and 48 h. IC_50_ values of TD in Huh7 cells after 24 and 48 h of exposure were determined as 100 μg/ml, which is used as an effective concentration for subsequent assays. As shown in the Figure 1a, TD had a marked dose dependent inhibitory effect on viability of Huh7 cells. TD was found to be less cytotoxic towards the normal liver cells of Chang liver cell line (Figure 1b).

### Trypan blue staining

**Figure 2:**
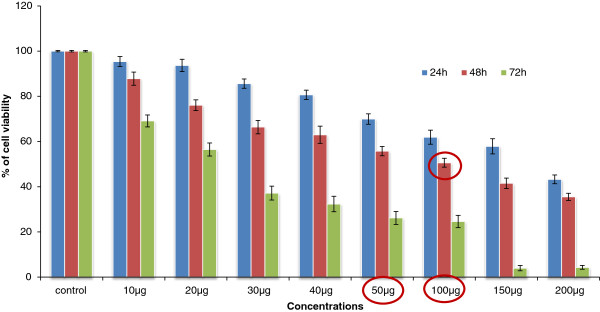
**Time and dose dependent effect of TD on antiproliferative (based on time kinetics) in Huh7 cells by Tryphan blue exclusion assay.** Values are expressed as mean ± SD for three independent experiments.

Viability of Huh7 cells treated with TD was assessed by trypan blue assay which showed a more effective action after 48 h treatment when compared to 24 and 72 h treatment (Figure 2). Huh7 cells when treated with TD, showed cell death at the end of 48 h. In the present study, the aqueous extract of TD induced cytotoxicity to Huh7 cells in a dose and time dependent manner. The results indicate that exposure of Huh7 cells to TD triggers the pathway of apoptosis leading to decrease in viability of Huh7 cells.

### Morphological analysis

**Figure 3:**
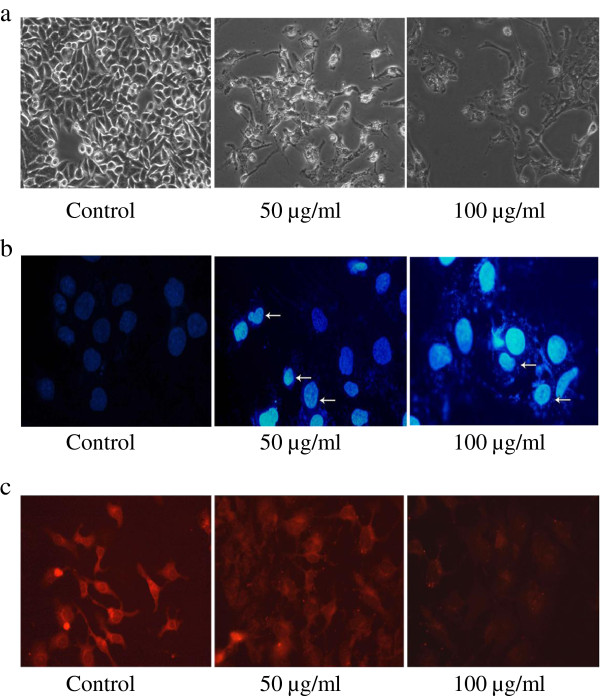
**Effect of TD on control and treated Huh7 cells. (a)** Morphological changes of Huh7 cells in the presence or absence of TD. Huh7 cells were treated with TD at the concentrations of 50 and 100 μg/ml for 48 h (magnification x40). **(b)** Nuclear changes after treatment with TD (50 and 100 μg) for 48 h, the Huh7 cells were stained with DAPI. **(c)** Huh7 cells were treated with 50 and 100 μg/ml of TD for 48 h, stained by Rh 123.

Microscopic analysis of Huh7 cells when treated with TD showed a decrease in cell number along with characteristic structural changes including vacuolization of the cytoplasm, which was not observed in Huh7 cells that were not exposed to treatment. TD reduced cell growth and dispersed the cells into individual, rounded cells (Figure 3a). These results further emphasize that the combination of herbs in TD might have caused the destruction of tumor cells.

### Nuclear staining

To assess the induction of apoptosis by TD, the cells were stained with DNA-specific (marker) fluorescent dye DAPI (4-, 6-diamidino-2-phenylindole) and examined by fluorescent microscopy. Huh7 cells, after treatment with TD (50 and 100 μg) for 48 h, were stained with DAPI nuclear stain for 5 min. As the cell membrane is compromised in apoptosis, more DAPI enters the cell and stains with a stronger blue color. The Huh7 cells treated with TD exhibited condensed and fragmented nuclei, indicative of apoptosis, as compared with the control cells which showed clear round nuclei (Figure 3b).

### Determination of mitochondrial membrane potential (Δψm) in cells treated with TD

Change in ΔΨm is considered to play a vital role in apoptosis. Loss or decrease in ΔΨm is followed by opening of mitochondrial permeability transition pores, which channel the leakage of cytochrome c and proapoptotic proteins from mitochondria into the cytosol. Cells were treated with 50 and 100 μg/ml of TD for 48 h, stained by Rh 123 and analyzed by fluorescence microscopy. Figure 3c shows the mitochondrial membrane potential in control and treated groups. Untreated Huh7 cells stained stronger for Rh123 with intact mitochondrial membrane, whereas TD treated cells stained less for Rh123 dye, indicating the loss of mitochondrial membrane potential.

### DNA fragmentation

**Figure 4:**
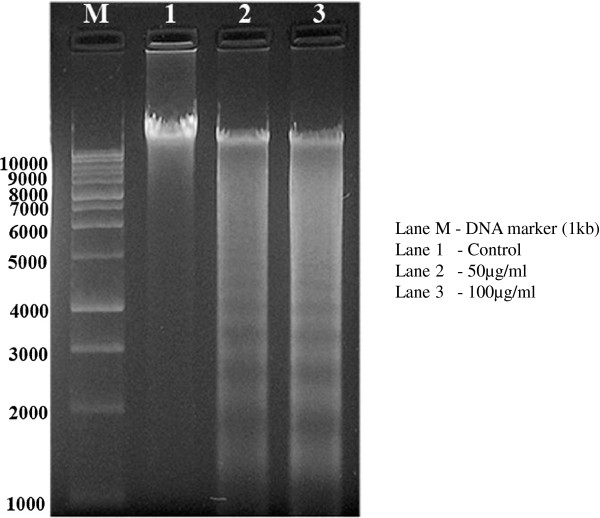
**DNA fragmentation of Huh7 cells induced by TD.** Cells were treated with TD at the concentrations of 50 and 100 μg/ml for 48 h. Fragmented DNA was isolated as described in Materials and methods and electrophoresis was performed in a 1.5% agarose gel.

Genomic DNA was isolated from treated and untreated Huh7 cells. Figure 4 depicts the agarose gel electrophoresis of DNA from Huh7 cells treated with TD. The Huh7 cells showed DNA fragmentation with a ladder pattern, which is a distinguishing aspect of apoptosis. These studies support the possibility that the cytotoxicity of TD might be through enhanced apoptosis.

### Mechanism of apoptosis

#### Modulation of Bad and Bcl-2/Bax ratios by TD treatment

**Figure 5:**
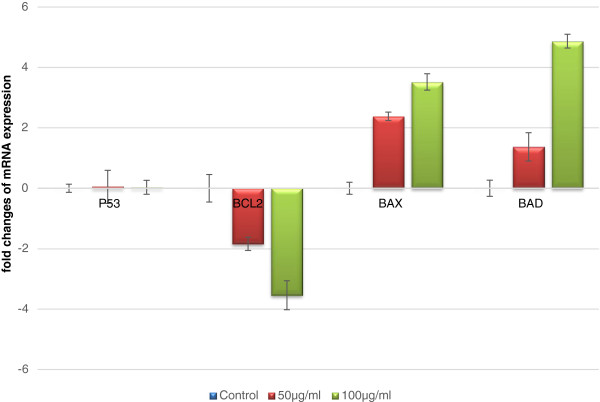
**Quantitative evaluation of mRNA expression of p53, Bcl2, Bax and Bad in control and treated Huh7 cells by Real-time PCR.** The cells were treated with TD at the concentrations of 50 and 100 μg/ml for 48 h. Relative expression levels of mRNA were expressed as fold changes. Values are the mean ± SD of data from three independent experiments.

**Figure 6:**
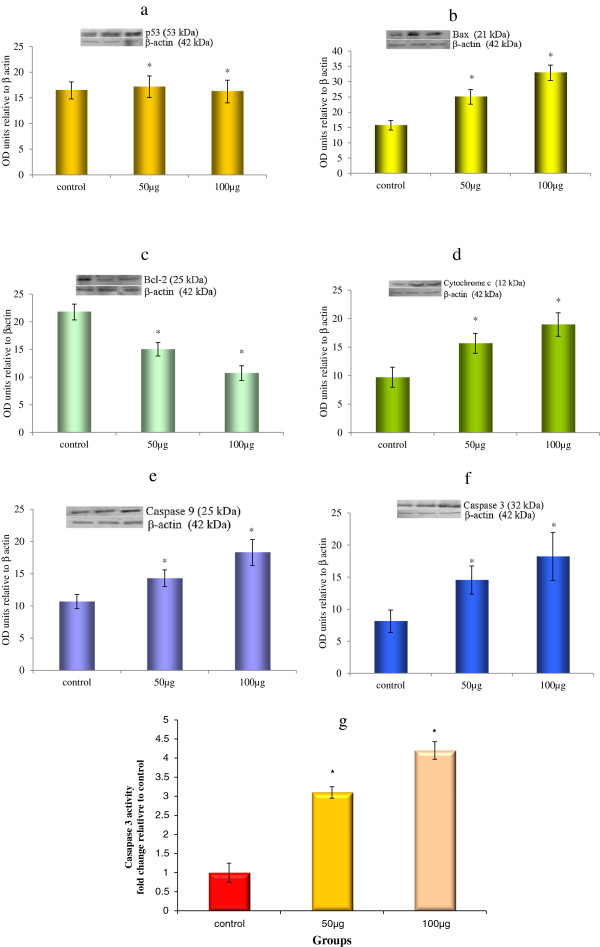
**Effect of TD on protein expression of p53, Bcl2, Bax, cytochrome C, Caspase 9 and Caspase 3 in control and treated Huh7 cells.** Evaluation of protein expression of p53 **(a)**, Bax **(b)** Bcl-2 **(c)**, cytochrome c **(d)**, Caspase 9 **(e)** and Caspase 3**(f)**. The cells were treated with TD at the concentrations 50 and 100 μg/ml for 48 h. Relative expression levels of proteins were expressed as the relative intensity of the bands. Evaluation of Caspase-3 activity in Huh7 cells by colorimetric method **(g)**. Values are the mean ± SD of data from three independent experiments.

Figure 5 portrays the Bcl-2, Bax and Bad expression by Realtime PCR. The levels of protein expression of Bcl-2 and Bax as determined by Western blotting analysis are depicted in Figure 6. In TD treated Huh7 cells, the expression of Bax and Bad were found to be up regulated, while expression of Bcl-2 was down regulated. Untreated cells show a basal expression of these proteins. Protein levels were quantified using densitometry analysis and are expressed in relative intensity arbitrary unit. These results suggest that TD could have induced apoptosis via alteration of the Bax/Bcl-2 ratio in Huh7 cells.

#### Effect of cytochrome c release into mitochondria

For analyzing the mitochondrial release of cytochrome c, proteins were prepared from cytosolic fraction and analyzed using Western blot. Cells exposed to TD treatment at 50 and 100 μg/ml concentrations for 48 hrs showed an increase in the expression of cytochrome c levels as compared to untreated cells (Figure 6). The loss of MMP observed in our study probably could have led to the release of cytochrome c into the cytosol. These results indicate that TD induces apoptosis at the mitochondrial level.

#### Induction of apoptosis by activation of caspase-3

Activation of caspases, a family of cysteine proteases, plays a central role in the execution of apoptosis. Western blot and colorimetric analyses of caspase-3 were carried out to investigate whether TD induced apoptosis via caspase activation. Expression of Caspase 3 gene was found to be up regulated in both Western blot (Figure 6f) and colorimetric assay (Figure 6g) of TD treated Huh7 cells in a dose dependent manner. The caspase-3 activity in Huh7 cells, as estimated by colorimetric assay, was up regulated to 2.01-fold and 3.2 fold at 50 and 100 μg/ml of TD, respectively. Furthermore, similar activation profile of caspase-9 was also observed. These results indicate that TD might have reduced Huh7 cell viability by activation of the caspase cascade.

#### Unaltered expression of p53

Treatment with TD does not influence the expression of p53 in Huh7 cells as there was no significant change in the levels of p53 mRNA (Figure 5) or protein (Figure 6) when compared to untreated control. In the present study, our results indicate that TD could have promoted apoptosis through activation of the caspase cascade, independent of p53 expression. This caspase activation would probably have resulted from increased levels of other proapototic mediators like Bax, as evidenced by its increased expression after treatment with TD.

#### Cell cycle analysis

**Figure 7:**
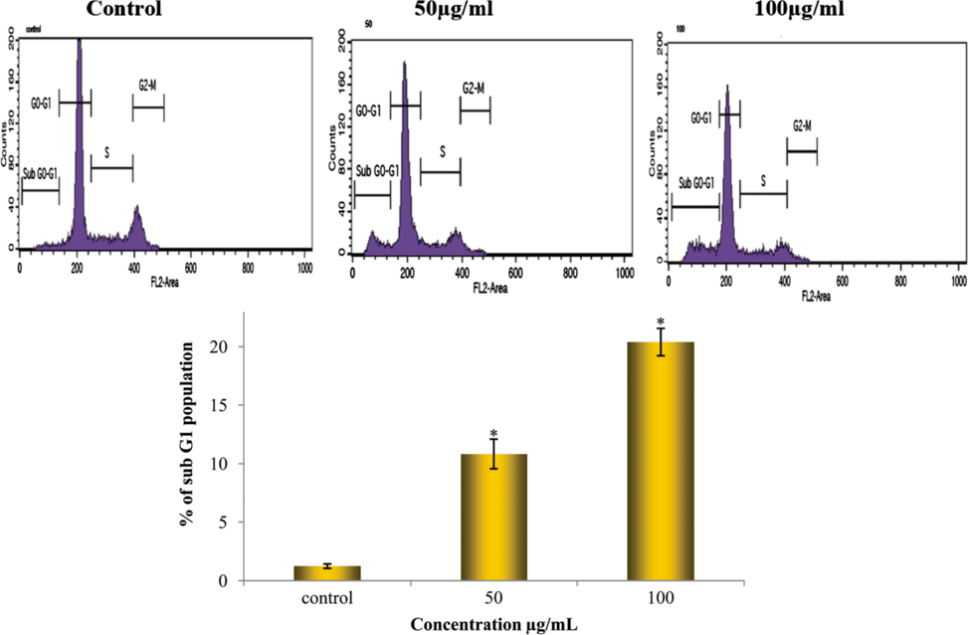
**Cell cycle phase analysis by flow cytometry in Huh7 cells treated with TD.** The cells were incubated for 48 h with medium alone (control), 50 and 100 μg/ml of TD. A graphic representation of the concentration-dependent increase in the percentage of apoptotic cells following TD treatment. Results are expressed as a percentage of the sub-G_1_ population obtained from the cells of flow cytometry data of propidium iodide fluorescence as described in Materials and methods, mean ± SD (n =3). Statistical significance *P < 0.05 as compared to the control, in the absence of TD.

Huh7 cells treated with TD were analyzed by flow cytometer which detects early changes in apoptosis and provides a lucid picture on the nature of cell cycle progression. The cell cycle distribution was examined at various times and indicated doses. The presence of apoptotic cells results in a peak at the sub-G1 position. It was noted that there was a remarkable accumulation of cells at the sub-G1 position (the so called sub-G1 peak) in TD treated Huh7 cells when as compared to control. Treatment of Huh7 cells with TD for 48 h led to profound changes of the cell cycle profiles (Figure 7). Treatment with TD at 50 μg/ml resulted in 10.83% of the cell population in the sub-G1 phase. However, when the cells were exposed to 100 μg/ml of TD, 20.4% of cells in the sub-G1 phase were observed. These results confirm the induction of apoptosis in Huh7 cells by TD in a dose dependant manner.

#### Immunofluorescence staining

**Figure 8:**
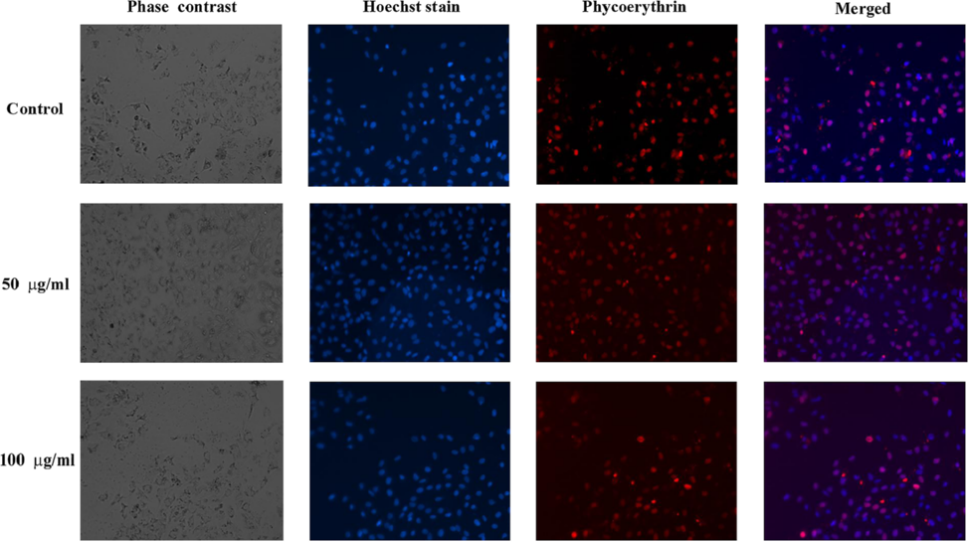
**Effect of TD on expression of Ki67 in control and treated Huh7 cells by Immunofluorescence staining.** Photomicrographs of the cells cultured with TD (control, 50 and 100 μg/ml) for 48 h shown in phase contrast, Hoechst stain (blue), Phycoerythrin (red) and merged.

Immunofluorescence staining was carried out for the purpose of observing changes in Ki-67 expression morphologically in the nucleus of the cells treated with TD. Untreated cells showed markedly increased expression for Ki-67 indicating high proliferative activity. TD treated cells showed negative nuclear expression for Ki-67 indicating low proliferative activity (Figure 8).

## Discussion

Hepatocellular carcinoma, one of the most malignant tumors with high mortality and poor prognosis, is well characterized by increased expression of antiapoptotic genes followed by rapid cell proliferation [42,43]. Previous studies show that various drugs used currently as chemotherapeutic agents in cancer, such as paclitaxel, vincristine, vinblastine, were identified through the study of various indigenous medicines [44]. However, instead of using individual herbal drugs for treating diseases with multiple indications, a combinational therapy is currently gaining greater importance [45]. The present study emphasizes the therapeutic efficacy of TD by demonstrating its cytotoxic potential in human hepatocellular carcinoma cell line (Huh7) and elucidating the cell death mechanism *in vitro.*

In the present study, TD was found to be cytotoxic and induced apoptosis in Huh7 cells in a time and dose dependent manner. The cytotoxic effect of the drug might be due to the synergistic effect of the three components of TD, *T. chebula*, *E. ganitrus* and *P. cineraria*. Previous studies have shown that TD and its components exerted cytotoxic activity against the human cancer cell lines HepG2, HepJ5 and HCT-15[26,38,46]. By MTT assay and trypan blue staining, we found that TD was significantly cytotoxic and decreased the viability of Huh7 cells. The microscopical analysis of Huh7 cells treated with TD also showed a decrease in cell number along with significant characteristic structural changes such as vacuolization of the cytoplasm when compared to untreated cells. Vacuolization and apoptosis of HeLa cells treated by nano selenium was reported by Huang *et al.,*[47]. Such vacuolization has been demonstrated to be related to selenium endocytosis [47]. Similarly vacuolization observed by us in Huh7 cells treated with TD could have resulted from endocytosis of the drug.

Apoptosis induction was confirmed by DAPI staining. TD treated cells exhibited condensed and fragmented nuclei, which is a hallmark of apoptosis. This finding indicates that TD causes cytotoxicity in Huh7 cells through a mechanism involving induction of apoptosis. Tumors rely on multiple signaling pathways to evade apoptosis and promote proliferation. Hence they are often resistant to chemotherapeutic agents which act on a single signaling pathway. However crude plant extracts may be more effective anticancer agents, as they have been found to activate multiple apoptotic pathways to combat malignant cells. Polyphenols, found in abundance in plant extracts, have been found to induce growth arrest and apoptosis in tumor cells [48]. Polyphenols have also been reported to inhibit or delay the cellular events associated with tumorigenesis, thus making them effective chemopreventive agents [49,50]. In agreement with these findings, TD which is a combination of herbs containing various polyphenols, alkaloids and flavonoids can activate multiple signaling pathways to induce apoptosis in tumor cells.

Apoptosis, a normal and widespread mode of cell death, is morphologically characterized by formation of apoptotic bodies, cell shrinkage, chromatin condensation, DNA fragmentation and membrane blebbing [51]. Loss of apoptosis in cancer cells is the key event for the process of cancer development. Malignant cells have been found reported to carry mutations in the apoptogenic genes such as p53, Bcl-2 family proteins or in genes encoding components of the caspase cascade [52,53].

In the present study, we observed that TD induced DNA fragmentation and DNA ladder formation, characteristic of apoptosis, in Huh7 cells [54]. Various phytoconstituents present in TD such as quercetin, chebulagic acid (found in *T. chebula*) [28] and palmitic acid (present in *E. ganitrus*) [32,33] might have contributed to the property of apoptosis induction.

We also conducted various molecular studies in order to evaluate the mechanistic action of the drug TD in Huh7 cells. The expression of Bax and Bad were found to be up regulated, while expression of Bcl-2 was down regulated in TD treated Huh 7 cells. This indicated that expression of these apoptosis related factors were regulated by TD in Huh7 cells. The translocation of Bax from cytosol to the mitochondrial membrane has been reported in response to various death stimuli [55-58]. We also observed an increase in Bax/Bcl-2 ratio following TD treatment. Previous studies have reported that increase in Bax/Bcl-2 ratio can cause mitochondrial membrane permeabilization, resulting in the release of cytochrome c and induction of apoptosis [59].

Increased levels of Bax protein have been shown to directly induce release of cytochrome c by forming a pore in the outer membrane of mitochondria [60]. Once released, cytochrome c couples with APAF-1 and dATP/ATP and forms a heptameric complex called the “apoptosome”, which then activates procaspase-9 [51]. Activated caspase-9 will then activate effector procaspases such as caspase-3,-6 and −7 to carry out the process of apoptosis [59]. Our study has shown an increased expression of cytochrome c in TD treated cells as compared to untreated cells. Change in ΔΨm measured by Rhodamine 123 staining have shown that TD treated cells showed loss of MMP which is consistent with the above findings. Mitochondrial permeability transition (MPT) that results from reduction and collapse of MMP is the major intracellular event which precedes the execution phase of the mitochondria mediated apoptosis pathway [61,62]. The above findings support the hypothesis that TD induces apoptosis by increased expression of Bax, thereby opening mitochondrial permeability transition pore (PTP) and cauding Δψm loss. The two latter events are the key signals for cytochrome c release from mitochondria into the cytoplasm. Cytochrome c then triggers caspase-mediated programmed cell death.

Caspases are a family of cysteine proteases which contain both upstream and downstream effector caspases. Activation of caspase cascade is solely responsible for the execution phase of apoptosis [63]. Caspase-3 is classically regarded as executor. Using western blot and colorimetric assay, we found that the expression of Caspase 3 was highly up regulated in TD treated Huh7 cells. Furthermore, a similar activation profile of caspase-9 was also observed. Thus, the activation of caspases-3 and-9 were involved in TD induced apoptosis of Huh7 cells. Treatment of Huh7 cells with TD could have led to loss of MMP, cytochrome c release through PTP and activation of caspases, eventually resulting in cell death.

p53, a tumor suppressor gene, is a well-established regulator of cell cycle arrest and is mutated in more than 50% of all tumors [64-66]. Mutation of p53 usually disrupts its DNA binding and transactivation functions. Therefore, mutant p53 is unable to negatively regulate cell growth following DNA damage, oncogene activation, hypoxia and loss of various normal cell contacts [52,67,68]. p53 restricts aberrant cell growth by cell cycle arrest at G1 or G2 phase or by the induction of apoptosis [69].

In the present study, Huh7 cells treated with TD showed no significant change in the levels of p53 mRNA or protein when compared to untreated control. In Huh7 cell line, TD was found to trigger apoptosis through activation of the caspase cascade, independent of p53 expression. The p53 gene in Huh7 cells, derived from a human hepatocellular carcinoma, has been shown to exhibit a point mutation at codon 220 [70]. This mutation renders the p53 gene product completely nonfunctional. Our findings were consistent with Hsu *et al.,*[70], who also observed a similar induction of apoptosis of Huh7 cells through enhanced activation of Bax and triggered caspase cascade, independent of p53 function. This result indicates that TD stimulates the mitochondrial mediated apoptotic pathway by increasing Bax/Bcl-2 ratio and activating caspase cascade, bypassing p53.

Cell cycle analysis was carried out by PI staining followed by flow cytometry in TD treated Huh7 cells. Presence of apoptotic cells can be identified by the presence of peaks in sub-G1 phase. In our study, we observed that the Huh7 cells treated with TD for 48 h led to profound changes in cell cycle profiles. We observed a remarkable accumulation of sub diploid cells within the sub-G1 phase. This result has strongly confirmed the induction of apoptosis in Huh7 cells following TD treatment. This apoptotic effect might be due to the synergistic activity of phenolic compounds such as flavonoids, tannins, gallic acid [71,72] and other active compounds in TD.

Ki-67 protein, which is associated with active cell proliferation, is found to be expressed in all phases of the cell-cycle, except G0. Highest levels of expression are observed in G2/M phase [73]. In this study, untreated cells showed markedly increased expression of Ki-67 indicating high proliferative activity in tumor cells. In a study of patients undergoing surgical resection for HCC, higher levels of expression of Ki-67 in tumor tissue were associated with higher tumor grade [73] and early disease recurrence [74]. Recent reports have proven that polyphenols decreased the expression of Ki-67 in cancer cell lines, OMC-4 and TMCC-1 [75]. Thus TD, which contains polyphenols, could have induced apoptosis in treated cells and decreased the expression of Ki-67.

## Conclusion

In conclusion, our findings suggest that TD is cytotoxic, with potent apoptotic activity in Huh7 cells. The apoptosis induction of TD in Huh7 cells is mediated via Bax-triggered mitochondria mediated pathway which activates caspase cascade, independent of p53 function. Gallic acid, which was isolated in highly purified form from TD and characterized, is a well-known phenolic antioxidant. The antioxidant and cytotoxic nature of TD might be partly due to the presence of gallic acid. Our findings collectively suggest that, in addition to isolation of gallic acid, various other active constituents of TD should be isolated and characterized to develop it as a potential therapeutic agent for treating hepatocellular carcinoma.

## Abbreviations

Huh7: Human hepatocellular carcinoma cell line; HCC: Hepatocellular carcinoma; TD: Tridham; MMP: Mitochondrial membrane potential; MOMP: Mitochondrial outer membrane potential; HCT-15: Human colon tumor cell line.

## Competing interests

There is no competing interest among the authors as far as this work is concerned.

## Authors’ contributions

PSh and PSa made substantial contributions to conception and design of the study. JR and RV carried out the experiments and analyzed the data. PSh and PSa revised the manuscript critically. All authors read and approved the final manuscript.

## Pre-publication history

The pre-publication history for this paper can be accessed here:

http://www.biomedcentral.com/1472-6882/13/323/prepub
